# Comparison of venous repair results using either arterial or vein grafts in a crush-avulsion injury model

**DOI:** 10.3906/sag-1808-38

**Published:** 2019-02-11

**Authors:** Selami Serhat ŞİRVAN, Işıl AKGÜN DEMİR, Fatih IRMAK, Dağhan DAĞDELEN, Kamuran Zeynep SEVİM, Ayşim ÖZAĞARI, Ayşin KARASOY YEŞİLADA

**Affiliations:** 1 Department of Plastic, Reconstructive, and Aesthetic Surgery, University of Health Sciences Şişli Hamidiye Etfal Research and Training Hospital, İstanbul Turkey; 2 Department of Plastic Surgery, Balıkesir State Hospital, Balıkesir Turkey; 3 Department of Pathology, University of Health Sciences Şişli Hamidiye Etfal Research and Training Hospital, İstanbul Turkey

**Keywords:** Microsurgery, replantation, amputation, arterial graft

## Abstract

**Background/aim:**

Venous insufficiency after replantation or revascularization is one of the most common causes of limb loss in either the short or the long term. The aim of this study was to evaluate the results of a new technique to overcome venous insufficiency.

**Materials and Methods:**

A crush-avulsion type of injury was formed in the femoral veins of rats of 3 separate groups. In the control group, primary repair was applied to the damaged veins and the remaining 2 groups were repaired with either an arterial graft or a vein graft. The success rates of anastomosis were then compared.

**Results:**

In the control group the patency rate was 25% in the 2nd hour, 12.5% on the 2nd day, and 12.5% on the 10th day. The patency rate in the vein group was 87.5% in the 2nd hour, 50% on the 2nd day, and 37.5% on the 10th day, whereas the patency rates in the artery group were 100% in the 2nd hour, 87.5% on the 2nd day, and 75% on the 10th day.

**Conclusion:**

Microsurgery requires experience and patience. It can be considered that the use of arterial grafts for venous repair in replantation after crush-avulsion type amputations can increase the success rate of replantation.

## 1. Introduction

As the number of traffic and occupational accidents increases, crush and avulsion types of amputations of the limbs have started to be seen more frequently. Due to increased microsurgical experience and recent developments in microsurgical technique, the success rate of replantation has increased. However, with greater industrialization, more crush and avulsion type injuries are seen rather than guillotine type, which have comparatively higher replantation success rates. Venous insufficiency after replantation or revascularization procedures is one of the most common causes of limb loss in either the short or the long term (1). If venous anastomosis is not performed during a replantation procedure, the success rate will be <20% (2). 

The hypothesis of this study was that the use of arterial grafts may be more successful in repairing a gap between veins that occurred after a crush-avulsion type of injury. Therefore, a crush-avulsion type injury model was created in rat veins. The damaged veins were repaired either primarily or with an arterial or venous graft and the success rates were evaluated and compared.

## 2. Materials and methods

**Figure 1:**
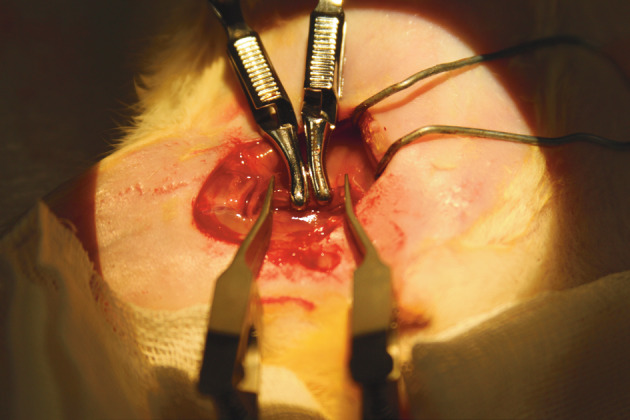
Crush-avulsion model.

Approval for this study was granted by the Üsküdar University Animal Research Ethics Committee. A total of 24 Sprague-Dawley rats, each weighing 250–300 g, were divided into 3 groups. Group 1 (8 rats) was designated as the control group, Group 2 (8 rats) the venous graft group, and Group 3 (8 rats) the arterial graft group. Anesthesia was administered intraperitoneally using ketamine hydrochloride at the dose of 50 mg/kg and xylazine hydrochloride at the dose of 10 mg/kg. The extremities of the rats were fixed to the operating table with tape, and the operative field was shaved and cleaned with an antiseptic solution. On each rat, a unilateral femoral incision was made and the femoral vein was dissected. To create a proper crush injury to the veins using a previously defined method, two Diffenbach bulldog clamps (maximum pressure 30 Nt, length 48 mm) were placed on the two ends of the femoral vein leaving a segment of 5 mm for 30 min (3). For the avulsion type injury model, a vascular approximator clamp was placed on the ends of the intentionally damaged segment of the vein and then the limbs of the approximator clamp were abducted from each other to stretch the vessel (Figure 1). In all groups, the crush-avulsion type injury was established and the microvascular clamps were placed to control bleeding in the next step. In the control group, the vein was directly cut and repaired primarily, without any excision. In Group 2, 0.5 cm of injured vein was excised and the gap was repaired with a venous graft of 1 cm taken from the external jugular vein. In the arterial graft group (Group 3), again a segment of 0.5 cm of the injured vein was removed and the gap was repaired with an arterial graft of 1 cm taken from the carotid artery. All anastomoses were performed by a single surgeon certified in advanced microsurgery. The patency of the anastomoses was evaluated in the 2nd hour and on the 2nd and 10th days using the milking test. 

On the 10th day after the patency assessment, the jugular vein was dissected in each rat and venous catheterization was performed. In order to visualize the femoral vein only, an opaque background was placed behind it. Indocyanine green was administered through the jugular catheter at a dose of 0.25 mg diluted in 0.01 mL of sterile water. Angiographic images were captured via the SPY Intraoperative Perfusion Assessment System (Lifecell Corp., Branchburg, NJ, USA). The vessel segments containing the anastomosis line were then excised and sent to the laboratory for histological examination including the evaluation of the endothelial damage and the ratio of intravascular thrombus diameter to that of the vessel.

Statistical analyses were performed using SPSS 15.0 for Windows. For the categorical variables, descriptive statistics were given as numbers and percentages. The ratios in the groups were compared with chi-Square analysis. Statistical significance was accepted as P < 0.05.

## 3. Results

**Figure 2:**
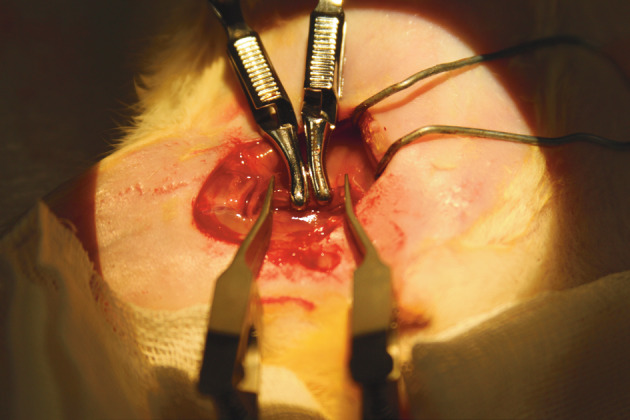
Control group. The 2nd hour (a), the 2nd day (b), and the 10th day (c) after anastomosis. Indocyanine green angiography appearance (d) on the 10th day.

**Figure 3:**
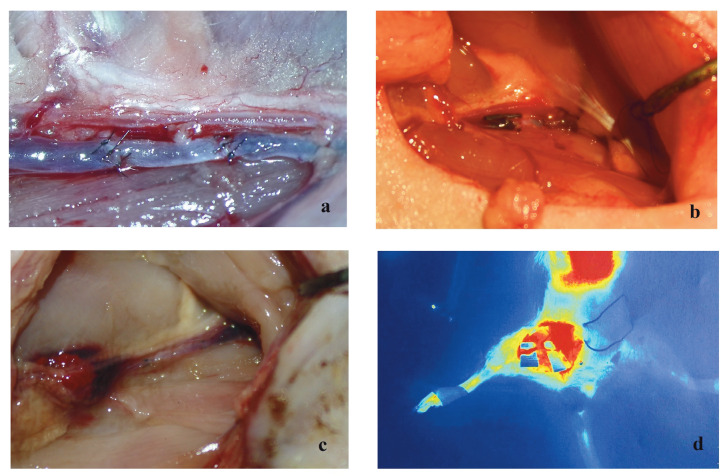
Venous graft group. The 2nd hour (a), the 2nd day (b), and the 10th day (c) after anastomosis. Indocyanine green angiography appearance (d) on the 10th day.

**Figure 4:**
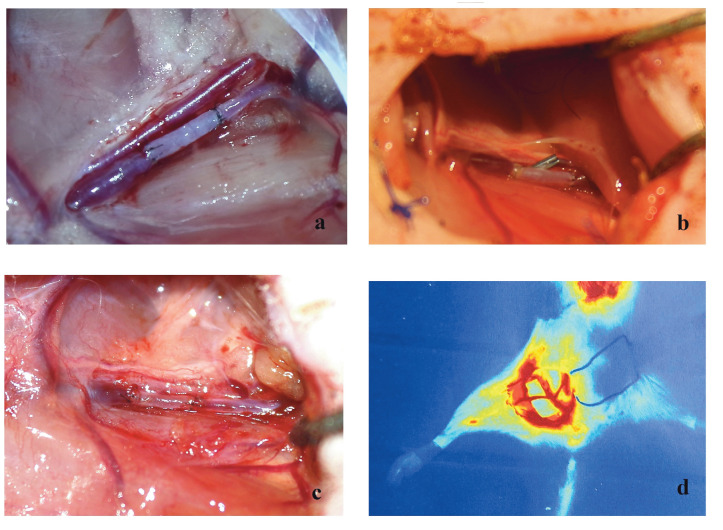
Arterial graft group. The 2nd hour (a), the 2nd day (b), and the 10th day (c) after anastomosis. Indocyanine green angiography appearance (d) on the 10th day.

**Figure 5:**
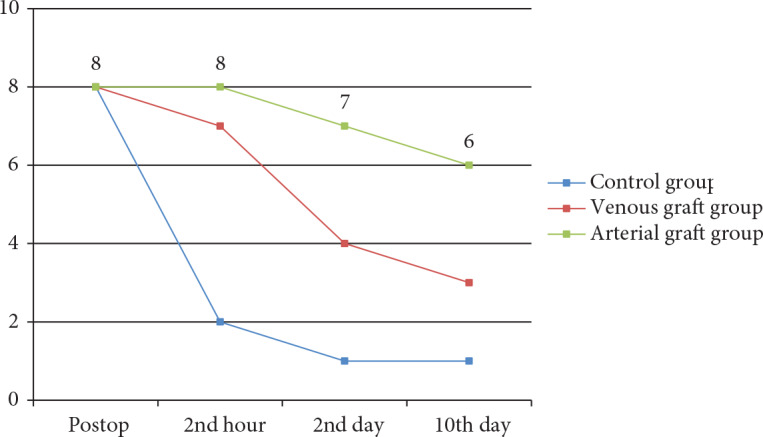
The evaluation of the anastomoses in the 2nd hour, on the 2nd, and on the 10th day.

Patency was observed in all groups immediately after the anastomosis. In the control group the patency rate was 25% in the 2nd hour, 12.5% on the 2nd day, and 12.5% on the 10th day. The patency rate in the vein group was 87.5% in the 2nd hour, 50% on the 2nd day, and 37.5% on the 10th day (on the first day of the study one rat died in the venous graft group), whereas the patency rates in the artery group were 100% in the 2nd hour, 87.5% on the 2nd day, and 75% on the 10th day (Figures 2–4). On the 10th day of the study angiography with indocyanine green was performed and the angiographic findings were seen to be consistent with the milking test results, showing patency in 1 rat in the control group, 3 rats in the venous graft group, and 6 rats in the arterial graft group (Figure 5).

### 3.1. Histological examination

Tissue samples were fixed with 10% neutral buffered formalin solution. After routine processing, the samples were embedded in paraffin wax. Tissue slices of 4 µm in thickness were stained with hematoxylin and eosin for evaluation with light microscopy.

**Figure 6:**
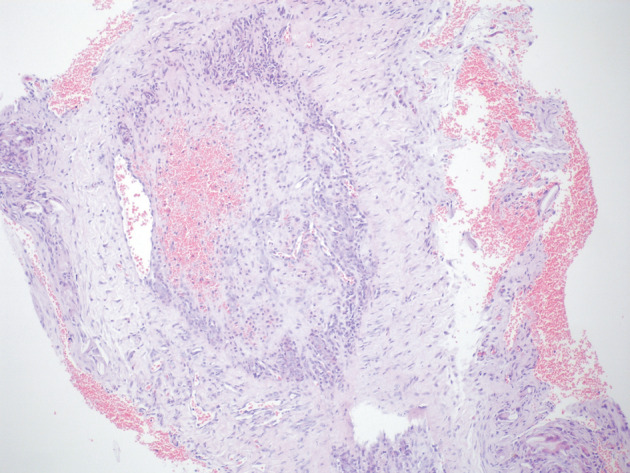
Histological image of the control group. Organized thrombus in the lumen. The lumen is occluded. Hematoxylin and eosin stain. Magnification 40×.

In the control group, tissues with diffuse crush artifacts were evaluated suboptimally. Organized thrombus and partial recanalization were observed in the vessels (Figure 6).

**Figure 7:**
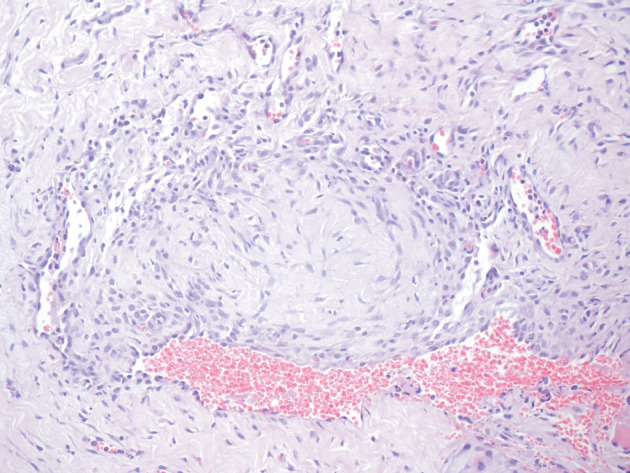
Histological image of the venous graft group. Organized thrombus in the lumen. The lumen is occluded. Hematoxylin and eosin stain. Magnification 40×.

In the venous graft group, the endothelial cells of the veins were damaged at both the proximal and distal parts of the anastomosis. Half of the tissue samples had occlusive thrombus in the vessels. Active inflammation and foreign body reaction in response to the suture material was observed in the surrounding tissue (Figure 7).

**Figure 8:**
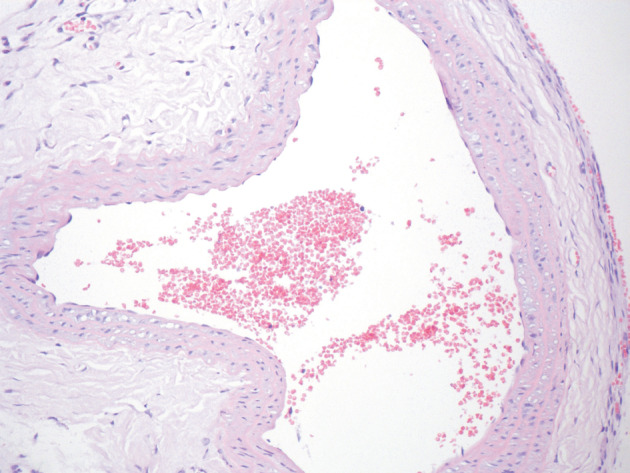
The histological image of the arterial graft group. The lumen is open. Minimally damaged endothelial cells. The internal elastic lamina had no pathological findings. Hematoxylin and eosin stain. Magnification 40×.

In the arterial graft group, minimally damaged endothelial cells were seen in the arteries including partial thrombus and recanalization. The internal elastic lamina of the arteries showed no pathological findings but one of the tissue samples showed necrosis in the tunica media. Organized thrombus and foreign body reaction in response to the suture material are shown in Figure 8.

In the analysis of the subgroups, the patency rates of the arterial graft group in the 2nd hour and on the 2nd and 10th day were significantly higher compared to the control group (P = 0.007, P = 0.010, P = 0.041). There was no statistically significant difference between the patency rates of the venous graft group compared to those of the control and arterial graft groups.

## 4. Discussion

Crush and avulsion type amputations are frequently encountered in the field of plastic surgery. Despite the advanced microsurgery techniques used in these particular types of injuries, thrombus formation may be seen at the anastomosis line resulting in failure of replantation (3). Saha et al. reported that venous insufficiency after replantation or revascularization procedures is one of the most common causes of limb loss in either the short or long term (1) and Tamai et al. stated that if venous anastomosis is not performed during the replantation procedure, the success rate would be below 20% (2). Narasimhan et al. emphasized that it may not always be possible to provide sufficient venous anastomosis in amputations at distal levels, in children, in cases of amputation with serious dorsal injury, and in cases where venous thrombus formation occurs after replantation (4).

Debridement of the nonviable tissues before starting replantation is mandatory in all cases of contaminated and crush-avulsion type injuries. First, there should be aggressive resection of the anastomosis segments that are thought to have endothelial damage to prevent thrombus formation in the postoperative period. It used to be believed that the endothelium functions as a passive barrier between blood and the vessel lumen, but recent studies have shown that local secretions of endothelium have a dynamic role in inflammation and thrombus formation (5). As a result of endothelial damage, the subendothelial layer is exposed, and when blood comes in contact with this layer, it causes platelet aggregation leading to thrombus formation (4). Clinically, endothelial damage results in vasospasm, thrombus formation, and atherosclerosis or restenosis (6). In most cases it is hard to define the rate of vascular damage (3). In a study by Mitchell et al. where an avulsion type injury model was created in rat extremities, the injured segment was measured as 0.8 cm under microscopy. However, when examined histologically, the actual damage was measured as up to 4 cm long (7). In distal amputation cases, considering the length of a finger, it is almost impossible to perform such an aggressive debridement. Narasimhan et al. suggested that vein grafts should be used routinely when there is a gap between the vessel ends after debridement or when there is tension in the anastomosis line after primary repair (4). A study by Shukla et al. regarding vein graft application showed that the tunica media of the vein graft became thicker and neointimal formation occurred due to the migration of vascular smooth muscle cells (8). Since the neointimal formation has an atherogenic feature, it may lead to plaque rupture and to occlusion of the graft. Vein grafts are also utilized frequently in coronary artery bypass procedures. Total vein graft loss secondary to thrombosis may be seen in up to 18% of patients in the short term (first 30 days), and up to 20%–50% in the medium and long term (9). There are many studies in the literature about the prevention of thrombus formation in vein grafts (3,10–12). 

When there is a gap between the vessel ends, synthetic grafts may also be utilized as an alternative to vein grafts. The ideal synthetic graft should be resistant to thrombus and aneurysm formation, to infection, and to ectopic calcification. It should also allow new vascular formation without neointimal hyperplasia or stenosis, while being easily obtainable and suitable for surgery, and should have similar features to those of a natural vessel (13). However, their use is limited to vessels with a diameter of 6 mm or more, since below that size vessels often tend to be occluded (14). The major advantage of synthetic grafts is that they do not cause any donor site morbidity. However, the disadvantages are the lack of an endothelial layer and local antithrombotic secretions.

Sirvan et al. reported the case of a patient who presented with bilateral total foot amputation at the proximal ankle level with a MESS score of 7. The patient had undergone below-the-knee amputation on one side, while the contralateral foot had been replanted successfully. During the replantation procedure, all the gaps between the veins and arteries were repaired with arterial grafts taken from the iatrogenically amputated segment of the leg (15). Saha et al. used interpositional arterial grafts to repair the gaps between the veins in 9 replantation cases, 6 being caused by crush type injury, and 8 of the replantation procedures were reported as successful (1). Despite reports of clinically successful results, no comparative experimental study regarding arterial grafts has been published as of yet. 

It has been suggested that the superficial localization of the veins makes them prone to injury and more susceptible to spasm because of increased sensitivity to temperature. In addition, as there is no tunica media in the veins and their walls have a fragile structure, the sustainability of patency is at stake, which may contribute to venous insufficiency (1). Veins are more likely than arteries to be affected by vasoactive substances (16). The vasa vasorum is the only blood supply to the veins, whereas arteries are supplied by both the vasa vasorum and their lumens (17). The endothelium of an artery releases more relaxing factors than a vein does (18), and the arterial wall can withstand higher blood pressures (1). Rockwell et al. reported that arterial grafts are more likely to secrete prostacyclins when compared to venous grafts within the postoperative 3-week period, which provides antithrombogenic activity (19). Due to their thin structure, edema resulting from trauma may likely interrupt the function of veins by applying pressure. Difficulty in microsurgical technique is another handicap caused by the absence of the tunica media layer in veins (20). Endothelial damage is seen rarely in the anastomosis lines of arterial grafts, whereas extensive dehiscence of the endothelial layer is observed in the same region of vein grafts within a couple of hours after the anastomosis is performed and renewal may take up to several weeks (21). Even if the endothelial layer is restored, the newly formed endothelium is highly dysfunctional (22).

In this study, the patency rate was significantly higher in the arterial graft group when compared to the control group, whereas the endothelial damage was less compared to both the control and venous graft groups. Since the number of subjects of this study was determined by the veterinarian of the local ethics committee according to the 3R rules, the limited number of subjects precluded making a significant comparison between the arterial graft and venous graft groups.

The major handicap of using arterial graft is the donor site morbidity. In crush-avulsion type injuries, where the arteries are unsuitable for anastomosis, the arteries can be dissected more proximally, excluding the injured segment, and these arterial grafts can be utilized as interpositional grafts, as in the cases described by Sirvan et al. (15) and Saha et al. (1). If all the arterial structures are suitable for anastomosis in a patient, this requires an additional donor site. The subscapular arterial system can be used for this purpose, providing grafts of up to 12 cm (1) without causing any problems other than a scar. The availability and applicability of arterial grafts have been reported in many studies utilizing internal mammarian artery, radial artery, posterior interosseous artery, inferior epigastric artery, thoracodorsal artery, and subscapular artery (19). Gastroepiploic artery, anterior interosseous artery, superficial inferior epigastric, and dorsalis pedis artery are among the other donor site options (23). However, the use of these arteries might decrease the number of options for free flap surgeries in the long term and the tissues supplied by these arteries might be predisposed to ischemia to a certain extent. In an assessment of 9 microsurgeons, Khouri concluded that the most important factor for a successful result is the experience of the surgeon (24).

In conclusion, microsurgery and replantation are procedures that require patience and technical skills. Not only the surgery but also the postoperative period including the patient follow-up should be handled meticulously. The success rate of replantation continues to increase due to developments in technology and increased microsurgical experience. It can be considered that repairing gaps between the vein ends with arterial grafts will increase the success rate of replantation after crush-avulsion type injuries.
